# Discordance between initial procalcitonin and Sequential Organ Failure Assessment score predicts in-hospital mortality in culture-positive bacterial sepsis: a retrospective cohort from an Indonesian tertiary hospital

**DOI:** 10.1016/j.ijregi.2026.100987

**Published:** 2026-08-27

**Authors:** Suriana Dwi Sartika, Risna Halim, Sudirman Katu, Ariantin Ulfah

**Affiliations:** 1 Division of Tropical and Infectious Diseases, Department of Internal Medicine, Faculty of Medicine, Universitas Hasanuddin, Dr. Wahidin Sudirohusodo General Hospital, Makassar, Indonesia; 2 Department of Oral Medicine, Faculty of Dentistry, Universitas Hasanuddin, Makassar, Indonesia

**Keywords:** Sepsis, Procalcitonin, SOFA score, Biomarker discordance, Mortality, Indonesia

## Abstract

**Objectives:**

A single initial procalcitonin (PCT) measurement has inconsistent prognostic value in sepsis. We examined whether a PCT that is low relative to the Sequential Organ Failure Assessment (SOFA) score identifies a distinct high-mortality phenotype in culture-positive bacterial sepsis.

**Methods:**

In this retrospective cohort of 441 adults with culture-positive bacterial sepsis at an Indonesian tertiary hospital (January 2023-June 2025), SOFA (≥6) and initial PCT (<2 ng/mL) were cross-classified into four concordance–discordance phenotypes (reference: concordant-low). In-hospital mortality was analyzed by logistic regression adjusted for the Charlson Comorbidity Index, Gram category, and serum creatinine; a continuous discordance index was used as a sensitivity analysis.

**Results:**

Overall mortality was 68.9%. Mortality differed across phenotypes (*P* < 0.001): concordant-low 62.5%, discordant low SOFA/high PCT 49.2%, concordant-high 78.8%, and discordant high SOFA/low PCT 90.4%. The high SOFA/low PCT phenotype was independently associated with mortality (adjusted odds ratio 6.10; 95% confidence interval 1.92-19.36), as was the continuous index (odds ratio 1.74 per SD; 95% confidence interval 1.42-2.14). The effect was additive rather than synergistic.

**Conclusion:**

Organ dysfunction disproportionate to the PCT response identifies a very high-mortality phenotype in culture-positive sepsis, independent of comorbidity, Gram category, and renal function, offering an accessible marker for early risk stratification in resource-limited settings.

## Highlights

• Low procalcitonin relative to Sequential Organ Failure Assessment marks a ∼90% mortality sepsis phenotype.
• The effect is independent of comorbidity, Gram category, and renal function.
• A continuous procalcitonin-Sequential Organ Failure Assessment discordance index predicts mortality robustly.
• An accessible marker for high-risk sepsis in a resource-limited setting.
• Findings from a high-burden Indonesian tertiary sepsis cohort.

## Introduction

Sepsis is defined as life-threatening organ dysfunction arising from a dysregulated host response to infection, and it remains one of the leading causes of death worldwide. It accounts for an estimated 11 million sepsis-related deaths each year, with a burden that falls disproportionately on low- and middle-income countries [1,2]. Current guideline-directed care therefore places considerable emphasis on early recognition and prompt risk stratification [3]. This need becomes particularly evident in tertiary referral settings across Southeast Asia, in which case-fatality rates remain high and tools capable of identifying the highest-risk patients at the time of diagnosis are of substantial clinical value [4].

Risk assessment in sepsis draws on two conceptually distinct dimensions. The Sequential Organ Failure Assessment (SOFA) score quantifies the degree of physiological organ dysfunction and has been embedded within the current consensus definition of the syndrome [2]. Procalcitonin (PCT), by contrast, reflects the intensity of the infectious and inflammatory stimulus. It is a calcitonin precursor whose transcription from the CALC-1 gene is induced in extrathyroidal tissues by bacterial components and proinflammatory cytokines, including interleukin-1β, interleukin-6, and tumor necrosis factor-α [5,6]. Since these two markers capture different facets of the septic process, representing organ failure and inflammatory burden respectively, the relationship between them within an individual patient may convey prognostic information that neither dimension provides on its own.

Despite extensive investigation, a single initial PCT measurement has shown inconsistent prognostic performance. Contemporary syntheses have concluded that PCT alone is of limited prognostic value, performing more reliably when interpreted alongside clinical severity indices or examined as part of biomarker kinetics [[7], [8], [9]]. This interpretation is further complicated by renal function, since impaired clearance can raise circulating PCT independently of active infection [10]. An alternative approach is to examine the discordance between a biomarker and the accompanying clinical severity, for which sepsis pathophysiology offers a plausible mechanistic basis. A profound anti-inflammatory or immunoparalytic host response may fail to generate a PCT elevation proportionate to the degree of organ dysfunction, and this blunted response has itself been associated with poor outcomes [11]. Whether such discordance between initial PCT and SOFA carries prognostic meaning has not yet been examined in culture-positive bacterial sepsis, and data from Indonesian and broader Southeast Asian populations remain scarce [4].

We therefore hypothesized that organ dysfunction disproportionate to the PCT response, represented by a high SOFA score in the presence of a relatively low initial PCT, would identify a distinct high-mortality phenotype. The present study examined the association between initial PCT-SOFA discordance and in-hospital mortality in a retrospective cohort of patients with culture-positive bacterial sepsis at an Indonesian tertiary hospital, with particular attention to whether any observed association remained independent of illness comorbidity, Gram category, and renal function.

## Methods

### Study design and population

This retrospective cohort study was conducted at Dr. Wahidin Sudirohusodo General Hospital, a tertiary referral hospital in Makassar, Indonesia, and was reported in accordance with the Strengthening the Reporting of Observational Studies in Epidemiology statement [12]. Data were collected retrospectively from the medical records of patients with sepsis admitted during the period from January 2023 to June 2025. Adults (≥18 years) with culture-positive bacterial sepsis and a documented in-hospital outcome were eligible for inclusion. Of 475 records screened, three were excluded for a missing outcome and 31 for an absent initial PCT measurement, leaving 441 patients in the final analysis (Supplementary Figure S1). The primary outcome was in-hospital mortality.

### Variables and definitions

SOFA and initial PCT were each dichotomized. Initial PCT was defined as the first PCT concentration measured within 24 h of the diagnosis of sepsis. SOFA was dichotomized at ≥6, which represented the cohort median and also corresponded to the Youden-optimal mortality threshold and a clinically meaningful degree of multiorgan dysfunction, whereas PCT was dichotomized at 2 ng/mL, the established threshold below which a clinically significant systemic bacterial infection is less likely. These thresholds were prespecified on clinical grounds rather than derived to optimize the observed associations; in a confirmatory receiver operating characteristic analysis, the Youden-optimal SOFA threshold coincided with the prespecified value of 6 (area under the curve 0.70), whereas the 2 ng/mL PCT cutoff is an established diagnostic threshold. Cross-classification produced four phenotypes: concordant-low (low SOFA/low PCT, used as the reference), concordant-high (high SOFA/high PCT), and two discordant groups (low SOFA/high PCT and high SOFA/low PCT). As a sensitivity analysis, discordance was expressed as a continuous index, defined as D = z(SOFA) − z(log-PCT). Covariates comprised the Charlson Comorbidity Index [13], Gram category, and serum creatinine; the last of these was included because PCT is renally cleared and may be elevated by renal dysfunction independently of infection [10].

### Data handling and statistical analysis

The characteristics of survivors and non-survivors were compared using the Mann-Whitney U test and the chi-square test. Missing covariate data were infrequent: SOFA and the Charlson Comorbidity Index were essentially complete, whereas serum creatinine was missing in a small number of patients. Adjusted models used complete cases without imputation (model *n* = 433). Mortality across the four phenotypes was compared using the chi-square test, and crude and adjusted odds ratios were estimated by logistic regression, with the concordant-low phenotype as the reference and adjustment for the Charlson Comorbidity Index, Gram category, and serum creatinine. Multicollinearity among covariates was assessed using the variance inflation factor, and model calibration was evaluated with the Hosmer-Lemeshow goodness-of-fit test. Robustness was assessed using the continuous index D and a SOFA × log-PCT interaction term, and the incremental value was examined using the integrated discrimination improvement and the continuous net reclassification improvement with bootstrap confidence intervals [14]. A two-sided *P* < 0.05 defined statistical significance, and all analyses were performed in Python (statsmodels, scikit-learn, and Sci*P*y).

## Results

### Cohort characteristics

Among 441 patients, in-hospital mortality was 68.9%. Non-survivors had higher SOFA scores and more frequent septic shock and Gram-negative sepsis than survivors (Table 1). Notably, median initial PCT was lower in non-survivors (7.2 vs 15.6 ng/mL), whereas the proportion with PCT <2 ng/mL did not differ significantly between groups i*n* the unadjusted comparison.

**Table 1:** Baseline characteristics and comparison based on mortality outcomes.

Characteristic	Non-survivors (n = 304)^a^	Survivors (n = 137)	*P*^b^
Age, years	57.0 (47.0-66.0)	54.0 (45.0-65.0)	0.120
Male sex	180 (59.2%)	83 (60.6%)	0.867
SOFA score	6.0 (4.0-9.0)	4.0 (3.0-6.0)	<0.001
Initial procalcitonin, ng/mL	7.2 (1.4-36.1)	15.6 (2.3-46.4)	0.056
Procalcitonin <2 ng/mL	87 (28.6%)	29 (21.2%)	0.127
Serum creatinine, mg/dL	1.1 (0.7-2.2)	1.2 (0.7-2.7)	0.466
Charlson Comorbidity Index	4.0 (3.0-6.0)	4.0 (3.0-5.0)	0.069
Septic shock	141 (46.4%)	27 (19.7%)	<0.001
Gram-negative sepsis	242 (79.6%)	90 (65.7%)	0.003

Abbreviation: SOFA, Sequential Organ Failure Assessment.

a Values are median (interquartile range) or *n* (%).

b *P* values from the Mann-Whitney U test for continuous variables and the chi-square test for categorical variables.

### Discordance phenotype and mortality

**Figure 1:**
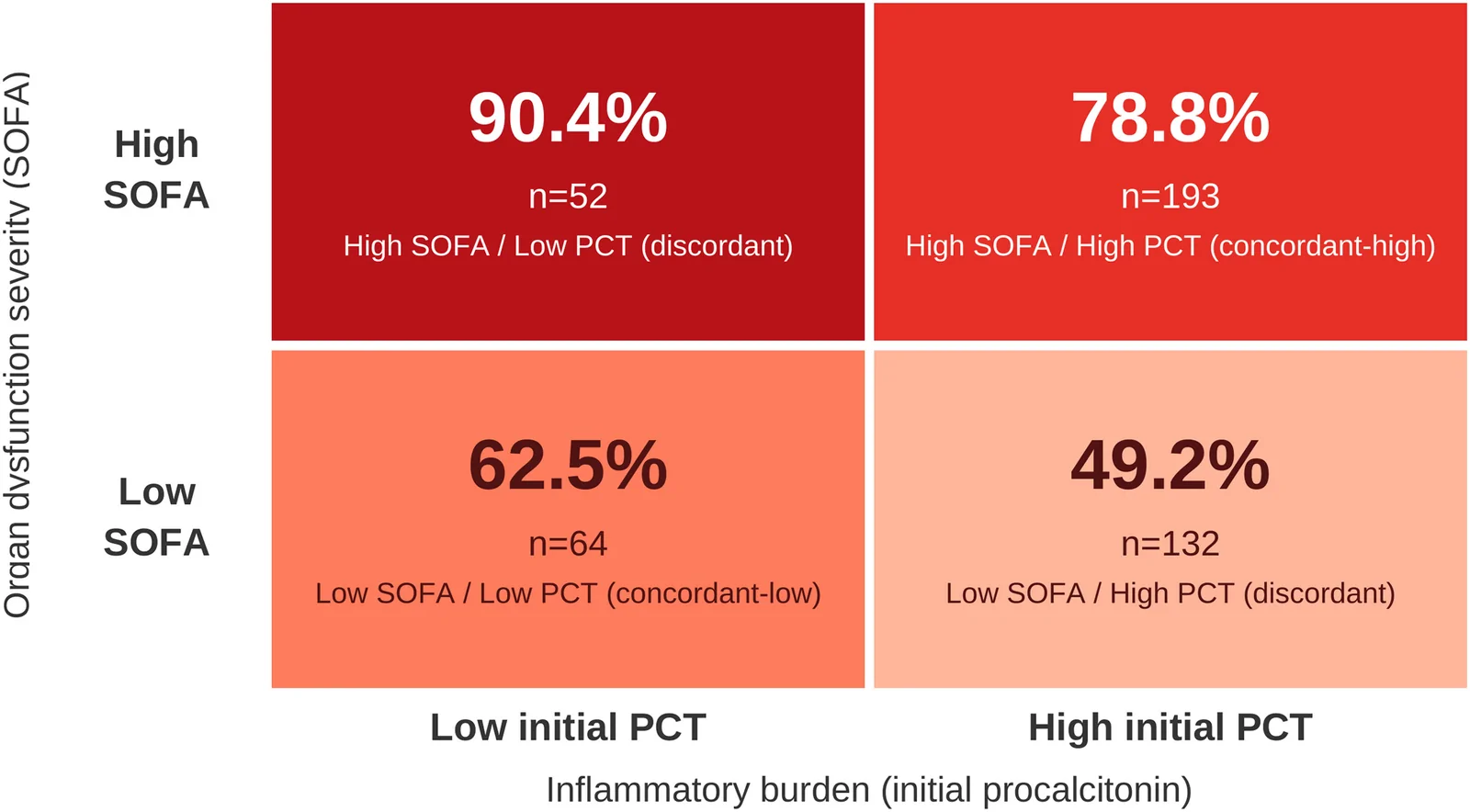
In-hospital mortality within each SOFA × initial procalcitonin phenotype. Values are the percentage of patients who died in hospital within each quadrant (not the distribution of patients across quadrants); cell sizes are given as *n*. PCT, procalcitonin; SOFA, Sequential Organ Failure Assessment.

Mortality differed markedly across the four phenotypes (chi-square *P* < 0.001). Relative to the concordant-low reference (62.5% mortality), the discordant low SOFA/high PCT group showed a trend toward lower mortality (49.2%; adjusted OR 0.58; 95% confidence interval [CI] 0.31-1.09), the concordant-high group had higher mortality (78.8%; adjusted OR 2.42; 95% CI 1.25-4.65), and the discordant high SOFA/low PCT group had the highest mortality (90.4%; adjusted OR 6.10; 95% CI 1.92-19.36; *P* = 0.002) after adjustment for comorbidity, Gram category, and creatinine (Table 2, Figure 1). Creatinine was not an independent predictor, indicating that the discordance signal was not attributable to renal function. The adjusted model (*n* = 433; area under the curve 0.724) showed no problematic multicollinearity (all variance inflation factors < 2.3) and adequate calibration on the Hosmer-Lemeshow test (*P* = 0.63).

**Table 2:** Association of PCT-SOFA discordance phenotypes with in-hospital mortality: bivariate and multivariable logistic regression.

Phenotype/covariate^a^	*n*	Deaths, *n* (%)	Crude OR (95% CI); *P*	Adjusted OR (95% CI); *P*^b^
Concordant-low (low SOFA/low PCT)	64	40 (62.5%)	1 (reference)	1 (reference)
Discordant (low SOFA/high PCT)	132	65 (49.2%)	0.58 (0.32-1.07); 0.082	0.58 (0.31-1.09); 0.089
Concordant-high (high SOFA/high PCT)	193	152 (78.8%)	2.22 (1.21-4.10); 0.011	2.42 (1.25-4.65); 0.008
Discordant (high SOFA/low PCT)	52	47 (90.4%)	5.64 (1.97-16.14); 0.001	6.10 (1.92-19.36); 0.002
Charlson Comorbidity Index (per point)	—	—	—	1.09 (1.00-1.19); 0.049
Gram-negative sepsis	—	—	—	1.69 (1.04-2.76); 0.034
Serum creatinine (per mg/dL)	—	—	—	0.94 (0.86-1.03); 0.192

Abbreviations: CI, confidence interval; OR, odds ratio; PCT, procalcitonin; SOFA, Sequential Organ Failure Assessment.

a Reference category: concordant-low phenotype.

b Adjusted for the Charlson Comorbidity Index, Gram category, and serum creatinine; model n = 433; area under the curve 0.724; all variance inflation factors < 2.3 (no multicollinearity); Hosmer-Lemeshow test *P* = 0.63 indicating adequate calibration.

### Sensitivity analyses

The continuous discordance index D was strongly and independently associated with mortality (adjusted OR 1.74 per SD; 95% CI 1.42-2.14; *P* < 0.001), confirming the finding without dichotomization and mitigating the small size of the high-risk discordant cell (*n* = 52). The SOFA × log-PCT interaction term was not significant, indicating an additive rather than synergistic effect. Improvement in reclassification metrics over a base model was small and not statistically significant (Table 3), consistent with a contribution to risk phenotyping rather than to global discrimination.

**Table 3:** Sensitivity analyses.

Sensitivity analysis	Result^a^
Continuous discordance index D = z(SOFA) − z(log-PCT), per SD, adjusted	OR 1.74 (95% CI 1.42-2.14); *P* < 0.001
SOFA × log-PCT interaction term	OR ≈1.01 (0.99-1.03); *P* = 0.52 (additive)
Integrated discrimination improvement (phenotype vs base model)	0.009 (95% CI −0.000 to 0.018); ns
Continuous net reclassification improvement	0.122 (95% CI −0.060 to 0.302); ns

Abbreviations: CI, confidence interval; ns, not significant; OR, odds ratio; PCT, procalcitonin; SOFA, Sequential Organ Failure Assessment.

a All estimates adjusted for the Charlson Comorbidity Index, Gram category, and serum creatinine.

## Discussion

This study demonstrates that, in bacterial sepsis, a low PCT concentration among patients with severe organ dysfunction constitutes a phenotype associated with in-hospital mortality. This association remained independent of comorbidity, Gram category, and renal function. The relationship was directional rather than symmetric. Among patients with a high SOFA score, a low PCT conferred the worst prognosis, whereas among those with a low SOFA score, a higher PCT was not deleterious and tended instead to be favorable. The continuous discordance index accounted for this association robustly, and the interaction analysis indicated that organ dysfunction and the PCT response contribute to risk in an additive rather than a synergistic manner.

Our findings are consistent with the observation that an initial PCT measurement has limited prognostic accuracy and acquires greater clinical meaning when it is assessed dynamically, for example as PCT clearance over time [[7], [8], [9]]. In the present study, the biomarker and the organ-dysfunction score were evaluated together at a single early time point, which allowed prognostic information to be obtained without the need for serial sampling. The analysis also accounted for the gram category of the infecting organism, since PCT concentrations tend to be higher in Gram-negative than in Gram-positive infection [6]. The discordance association nevertheless persisted after adjustment for Gram category, which indicates that the association is not explained by the broad microbiological category of the infecting pathogen.

The prognostic direction observed here is biologically coherent and can be understood through sepsis-induced immunosuppression. Under physiological conditions, PCT synthesis is minimal; during systemic bacterial infection, its expression is induced 100- to 1000-fold through proinflammatory cytokine stimulation, particularly by interleukin-1β, interleukin-6, and tumor necrosis factor-α [5,6]. An elevation in PCT therefore depends on the capacity of the host to mount a coordinated cytokine response. In patients with preexisting immunocompromizing conditions, such as malignancy, diabetes mellitus, or prolonged corticosteroid exposure, this innate response may be markedly blunted at sepsis onset, so that an adequate cytokine cascade cannot be generated despite progressive organ dysfunction. This state has been characterized as a profound immunosuppressive phenotype, in which immune and metabolic regulation fail to return to homeostasis [15].

This interpretation is reinforced by evidence challenging the classical sequential model of sepsis immunopathology, in which hyperinflammation was assumed to precede a compensatory anti-inflammatory response. More recent work indicates that immunosuppression can emerge concurrently with, or immediately after, the initial inflammatory insult, particularly in vulnerable hosts. This early immunoparalysis, defined operationally by monocyte human leukocyte antigen-DR expression ≤30%, is an independent predictor of in-hospital mortality, an association reported particularly among older patients [[16], [17], [18]]. Organ dysfunction in this phenotype is driven not only by cytokine-mediated injury but also by noninflammatory pathways. These include mitochondrial dysfunction and bioenergetic failure—impaired electron transport chain activity, reduced adenosine triphosphate synthesis, and increased reactive oxygen species—a mechanism termed cytopathic hypoxia that perpetuates cellular dysfunction independently of the systemic inflammatory signal [19].

The clinical consequence of this decoupling between organ failure and the inflammatory biomarker response is that the SOFA score accurately reflects the cumulative burden of organ dysfunction, whereas PCT fails to capture the severity of the underlying host response. A high SOFA score accompanied by a disproportionately low PCT therefore does not indicate a mild infection, but instead identifies a subgroup in whom standard biomarker-guided clinical assessment may systematically underestimate mortality risk [15,18]. This framework also explains two further features of our findings. It clarifies why the association persisted after adjustment for serum creatinine, since the phenomenon reflects impaired biomarker generation rather than altered renal clearance, and it explains why PCT becomes prognostically informative only when it is interpreted relative to clinical severity, because it is the discordance between the two dimensions, rather than the absolute concentration, that captures the immunological state of the host [7,8].

These findings arise from a tertiary referral cohort with high illness severity and a Gram-negative predominance; the absolute mortality within each phenotype is therefore likely to be lower in community hospitals and in populations with different pathogen distributions. Moreover, the applicability of the discordance approach is itself constrained by resource availability: PCT assays and complete SOFA scoring, which requires arterial blood gas, bilirubin, creatinine, and platelet measurements, are not uniformly accessible across Indonesian hospitals, particularly district-level facilities outside major urban centers. The phenotype-specific risks reported here should therefore be re-estimated in lower-acuity and epidemiologically distinct settings, and the approach itself validated where the required measurements are available, before clinical use.

This study has several strengths, including a well-characterized culture-positive cohort, the explicit adjustment for renal function that addresses the principal biological confounder of PCT interpretation, and the convergence of the categorical and continuous analyses upon the same conclusion. Some limitations should nevertheless be acknowledged. The study was retrospective and confined to a single center, which constrains generalizability. The high-risk discordant group was small, which widened the CI for its effect estimate and is the principal reason that the continuous discordance index, which uses all available information, was emphasized as the more stable measure. In addition, the high baseline mortality of the cohort limits both the negative predictive value and the global discriminatory performance of any single marker, and the gains in reclassification metrics did not reach statistical significance, indicating that the contribution of the discordance phenotype lies in risk phenotyping rather than in improved global discrimination. Finally, this study did not assess the timing of first antibiotic administration or the adequacy of antimicrobial therapy, both of which are recognized predictors of mortality in sepsis, and residual confounding by these factors therefore cannot be excluded. These findings should therefore be regarded as hypothesis-generating and warrant prospective, multicenter validation before clinical application.

## Conclusions

In patients with culture-positive bacterial sepsis, an initial PCT that was low relative to the degree of organ dysfunction identified a phenotype with markedly high in-hospital mortality, independent of comorbidity, Gram category, and renal function. This discordance, most robustly expressed as a continuous index, may serve as an accessible marker to flag patients whose blunted inflammatory response belies their clinical severity. Given the retrospective and exploratory nature of the study, these findings should be regarded as hypothesis-generating and warrant prospective, multicenter validation before clinical application.

## CRediT authorship contribution statement

**Suriana Dwi Sartika:** Writing – original draft, Methodology, Investigation, Formal analysis, Conceptualization. **Risna Halim:** Writing – review & editing, Supervision, Conceptualization. **Sudirman Katu:** Writing – review & editing, Supervision. **Ariantin Ulfah:** Investigation, Data curation.

## Declaration of competing interest

The authors have no competing interests to declare.

## Appendix Supplementary materials

**Supplementary Figure S1.** Study flow and phenotype classification. Image, application 1
