# Supplementary material for: Discordance between initial procalcitonin and Sequential Organ Failure Assessment score predicts in-hospital mortality in culture-positive bacterial sepsis: a retrospective cohort from an Indonesian tertiary hospital
Source: IJID Reg. 2026 Aug 27;21:100987. doi: 10.1016/j.ijregi.2026.100987 (PMC13587739; doi:10.1016/j.ijregi.2026.100987)
Supplement: Supplementary file 1 [file mmc1.docx]

**Supplementary Figure S1.** Study flow and phenotype classification
